# Salivary and serum levels of lactate dehydrogenase in oral submucous fibrosis: A meta-analysis

**DOI:** 10.1097/MD.0000000000037788

**Published:** 2024-04-12

**Authors:** Xueru Chen, Tongqiang Chen, Hui Xie, Jincai Guo

**Affiliations:** a Department of Pharmacy, Changsha Stomatological Hospital, Changsha, China; b Hunan Provincial Institute of Product and Goods Quality Inspection, Changsha, China.

**Keywords:** biomarker, diagnose, lactate dehydrogenase, oral submucous fibrosis

## Abstract

**Background:**

The occurrence of oral submucous fibrosis (OSF) is often accompanied by an increase in lactate dehydrogenase (LDH) levels. In this meta-analysis, we compared the salivary and serum levels of LDH levels between OSF patients and controls.

**Material and methods:**

A comprehensive search was conducted in PubMed, Embase, Web of Science, and Cochrane Library from the establishment of the database to June 2023, and the quality of the studies was checked by the Newcastle-Ottawa Quality Assessment scale. The mean difference (MD) and 95% confidence interval (CI) were calculated using RevMan 5.4 software.

**Results:**

A total of 28 studies were retrieved from the database, and we included 5 studies in this meta-analysis. The salivary LDH level of OSF patients was higher than healthy controls (MD: 423.10 pg/L 95%CI: 276.42−569.77 pg/mL, *P* < .00001), the serum LDH level of OSF patients was also higher than that of healthy controls (MD: 226.20 pg/mL, 95%CI: 147.71−304.69 pg/mL, *P* < .00001).

**Conclusions:**

This meta-analysis showed that salivary and serum LDH levels were higher in OSF patients than in healthy controls, suggesting that LDH may be a potential biomarker for OSF.

## 1. Introduction

Oral submucosal fibrosis (OSF) is a chronic, insidious, inflammatory and potentially malignant disease of oral mucosa with excessive tissue repair, and the malignant transformation rate is 4.2%.^[1]^ The disorder of collagen metabolism is the main pathogenesis. Disease occurs as a result of increased collagen formation and decreased degradation, which results in deposition of collagen fibers in oral tissues. The clinical manifestations become more and more severe with the progression of the disease, including burning sensation of spicy food, tongue dysmotility, ulcers, fibrosis of the buccal mucosa, and finally progressive limitation of mouth opening.^[2,3]^ The etiology of OSF is multifactorial, including vitamin and iron deficiency, immune processes, genetic susceptibility, use of pepper, betel nut, etc. Among them, betel nut chewing is the main reason.^[4,5]^

To understand the pathogenesis and identify clinically relevant biomarkers, many studies have explored the changes at the molecular level in the body fluids of OSF patients. There have been reports of OSF molecular biomarkers for pathogenesis and malignant transformation.^[6,7]^

Lactate dehydrogenase (LDH) is one of the most common enzymes in nature, which is the main component after glycolysis. It is present in the cytoplasm of body tissues and is a marker of inflammation.^[8,9]^ This enzyme catalyzes the conversion of glucose to pyruvate during aerobic glycolysis.^[10]^ The expression of LDH is important to patients with OSF, it is mainly reflected on some aspects as follows. Firstly, LDH is an enzyme found in the cytoplasm of cells. When cells are damaged, LDH is released into the extracellular space.^[11]^ Therefore, the change in LDH levels can reflect the tissue cell damage, which is very helpful in assessing the progress of patients with OSF. Secondly, LDH is an important enzyme in cellular energy metabolism. The change in LDH levels may reflect alterations in cellular metabolic activity during the disease process. As OSF is a chronic inflammatory disease, changes in metabolic activity are closely related to disease progression. Finally, studies have shown that inflammation is closely associated with the fibrotic process.^[12]^ LDH is an inflammatory marker and OSF is characterized by inflammation and fibrosis.^[13,14]^ Therefore, LDH may play an important role in the development of patients with OSF.

Analysis of serum and saliva can be a useful tool in the diagnosis and treatment of systemic diseases.^[15,16]^ Some studies have reported that the level of LDH in serum and saliva of OSF patients is high, however, whether LDH can be used as a diagnostic marker of OSF is unclear, so we conducted this meta-analysis. To the best of our knowledge, there is no meta-analysis of saliva and serum LDH levels in OSF, so we aimed to conduct a meta-analysis of LDH levels in saliva and serum in OSF.

## 2. Materials and methods

The reporting of the present meta-analysis is in accordance with the Preferred Reporting Items for Systematic Reviews and Meta-Analyses (PRISMA) protocols.^[17]^ This meta-analysis was registered with PROSPERO with the code CRD42023428860. The PECO

(Participants, Exposure, Control and Outcomes) question: “Are serum and salivary LDH levels different in OSF patients compared to healthy controls?” (P: humans with and without OSF at any age and sex; E: OSF disease; C: healthy controls; O: changes in the serum and salivary LDH levels).

### 2.1. Search strategies

We used Medical Subject Headings (MeSH) and free words to find search terms. The PECO terms were applied to derive keywords. We searched 4 databases (PubMed, Web of Science, Embase, and the Cochrane Library) from inception to June 3, 2023. The PubMed search strategy was as follows:

#1 “oral submucous fibrosis”[MeSH] OR “OSF”[MeSH] OR “OSMF”[MeSH]
#2 (((((((atrophia idiopathica mucosae oris [Title/Abstract]) OR (diffuse oral submucous fibrosis [Title/Abstract])) OR (submucous fibrosis of the palate and pillars [Title/Abstract])) OR (idiopathic scleroderma of the mouth [Title/Abstract])) OR (asian sideropenic dysphagia [Title/Abstract])) OR (sclerosing stomatitis [Title/Abstract])) OR (idiopathic palatal fibrosis [Title/Abstract])) OR (juxta-epithelial fibrosis [Title/Abstract])
#3 #1 OR #2
#4 lactate dehydrogenase [Title/Abstract]
#5 LDH[Title/Abstract]
#6 #4 OR #5
#7 #3 AND #6

### 2.2. Eligibility criteria

Inclusion criteria: case-control studies without any restrictions; studies reporting salivary and serum LDH levels in OSF patients and controls; clinical or pathological diagnosis of OSF were included in the study; OSF patients had no other systemic diseases.

Exclusion criteria: studies with incomplete data; studies including patients with multiple oral diseases; no full-text or abstract literature was available.

### 2.3. Study selection and data extraction

After a rough screening on the basis of title and abstract and then by reading the full text, articles were included using a prespecified data extraction form. In each step, 2 reviewers JG and XC independently screened the literature and extracted the data, then checked each other. If there were different opinions, the third reviewer HX would assist in the judgment.

### 2.4. Quality assessment

The quality of the studies was evaluated by 2 authors (XC and HX) using the Newcastle-Ottawa Scale (NOS) assessment^[18]^ with a maximum score of 9 for each study. The study quality was divided into 3 categories: high quality (scored 7–9); moderate quality (scored 4–6); and low quality (scored 0–3). The details of the study quality evaluation are shown in Table 1.

**Table 1:** Quality score of each study were entered to the meta-analysis.

The first author, publication yr	Selection (0–4)	Comparability (0–2)	Exposure (0–3)	Total score
Mishra, Silpiranjan, 2018	**	**	***	7
Mantri, T, 2019	***	**	***	8
Sivaramakrishnan, M, 2015	**	**	***	7
Kallalli, B. N, 2016	**	**	***	7
Panda, Abikshyeet, 2020	**	**	***	7

### 2.5. Statistical analysis

Statistical analysis was performed using RevMan 5.4 software. Continuous data were analyzed using the mean difference (MD) and 95% confidence interval (CI). Heterogeneity was evaluated by the inconsistency index statistic (*I*^2^). A fixed-effects model was chosen if no heterogeneity was observed (*P* > .1, *I*^2^ ≤ 50%). If statistical heterogeneity was present (*P* ≤ .1, *I*^2^ > 50%), further analysis of the source of heterogeneity was performed, after excluding the effects of significant clinical heterogeneity, a random-effects model was used for meta-analysis. The unit of LDH level in the analyses was picogram per milliliter (pg/mL).

### 2.6. Ethical approval

This meta-analysis does not require ethical assessment because only indirect literature will be included and evaluated.

## 3. Results

### 3.1. Literature search

**Figure 1.**
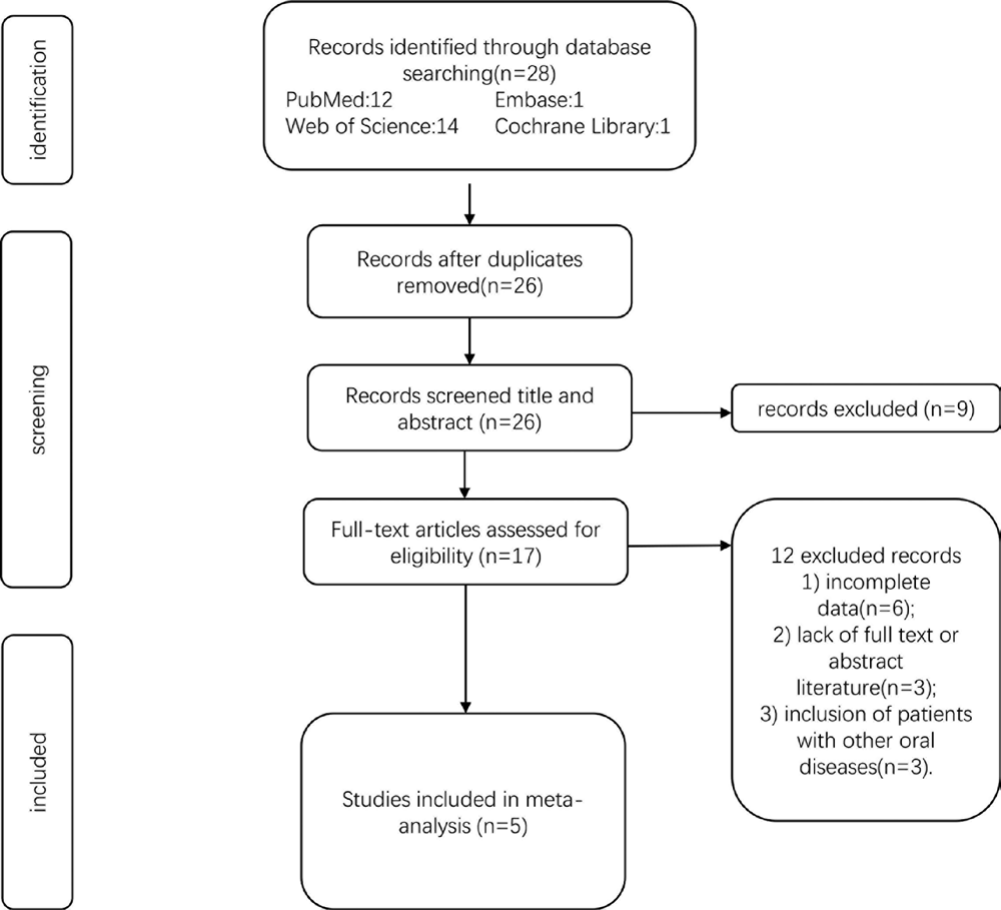
Flowchart of the study selection.

In total, 28 relevant studies were examined using our search strategy. Eleven duplicate studies were excluded. After the screening of the titles and abstracts of the remaining 17 articles. 12 articles were excluded due to incomplete data (n = 6), lack of full text or abstract literature (n = 3), and inclusion of patients with other oral diseases (n = 3). The detailed screening process is shown in Figure 1. Based on the inclusion and exclusion criteria, 5 reports published from 2015 to 2020 were ultimately included in meta-analysis.

### 3.2. General characteristics and clinical parameters

The characteristics of the selected studies included the name of the first author, publication year, and the baseline characteristics of the subjects, including sample size, age, and gender. Five studies included in this meta-analysis were reported from 2015 to 2020.^[15,19–22]^ Three studies reported both salivary and serum LDH levels,^[20–22]^ and 2 studies reported salivary LDH levels.^[15,19]^ All studies reported the participants from India. All studies included 145 OSF patients and 130 healthy controls. The general characteristics and clinical parameters of the included reports are shown in Table 2.

**Table 2:** Characteristics of the articles included in the meta-analysis.

The first author, publication yr	Country	Participants		Age (yr)		Gender (M/F)		Sample
		T	C	T	C	T	C	
Mishra, Silpiranjan, 2018	India	20	20	28.63 ± 10.39	26.20 ± 7.04	20/0	20/0	Saliva & Serum
Mantri, T, 2019	India	30	30	/	/	/	/	Saliva
Sivaramakrishnan, M, 2015	India	30	30	18–60	18–60	/	/	Saliva & Serum
Kallalli, B. N, 2016	India	25	10	20–70	20–70	/	/	Saliva
Panda, Abikshyeet, 2020	India	40	40	20–69	20–69	38/2	35/5	Saliva & Serum

### 3.3. LDH level in serum

**Figure 2.**
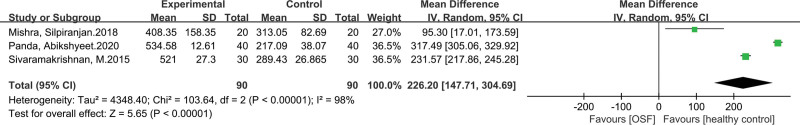
Forest plot of serum LDH levels in the OSF patients compared with the healthy controls. Serum LDH levels in the OSF patients were significantly higher than healthy controls. LDH = lactate dehydrogenase, OSF = oral submucous fibrosis.

This meta-analysis from 3 studies showed a significantly high level of LDH of serum in OSF patients compared with healthy controls (MD: 226.20 pg/mL, 95%CI: 147.71−304.69 pg/mL, *P* < .00001). However, statistically significant heterogeneity was found among these studies (*I*^2^ = 98%, *P* < .001) (Fig. 2).

### 3.4. LDH level in saliva

**Figure 3.**
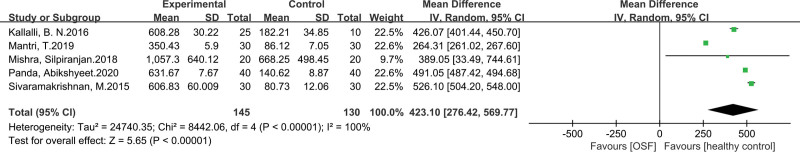
Forest plot of salivary LDH levels in the OSF patients compared with the healthy controls. Salivary LDH levels in the OSF patients were significantly higher than healthy controls. LDH = lactate dehydrogenase, OSF = oral submucous fibrosis.

The meta-analysis from 5 studies showed significantly high levels of LDH of saliva in OSF patients compared with healthy controls (MD: 423.10 pg/mL, 95%CI: 276.42−569.77 pg/mL, *P* < .00001). However, statistically significant heterogeneity was found among these studies (*I*^2^ = 100%, *P* < .001) (Fig. 3).

### 3.5. Quality evaluation

Table 1 shows the quality score for each study in the meta-analysis. The mean quality score of 1 study was 8,^[15]^ and other 4 studies were 7.^[19–22]^ The quality of all studies was high quality.

## 4. Discussion

OSF is a chronic disease of the ora66l cavity characterized by alterations in submucous fiber flexibility and a subepithelial inflammatory response. Almost all bodily tissues contain the enzyme LDH in cytoplasm. the presence of this substance outside of cells is invariably associated with tissue damage and cellular necrosis. There are increasing attempts to develop noninvasive and reliable biochemical tests for early detection and diagnosis. In the field of oral pathology, LDH activity assays are used to screen for oral diseases such as OSF,^[15,19–22]^ oral leukoplakia (OL),^[23,24]^ oral lichen planus,^[25]^ and oral cancer.^[26]^ To the best of our knowledge, this is the first meta-analysis to analyze LDH levels in the serum and saliva of OSF patients. This meta-analysis included 5 studies (145 OSF patients and 130 healthy controls), saliva was collected in the morning in all the subjects and they did not eat, drink, or smoke for at least 1 hour before collection, blood samples were collected under aseptic precautions by vein puncture. The results showed that saliva and serum LDH levels were significantly higher in OSF patients than in healthy people.

Research from Bhuvaneswari et al^[23]^ shows that saliva was collected from 20 healthy controls, 20 OL, and 20 oral squamous cell carcinoma, LDH levels were measured. The results showed that salivary LDH was increased in patients with OL and OSCC. It is proved that it is proved that salivary LDH may be a potential biomarker to identify early precancerous or malignant lesions. Kumar et al^[16]^ found that the levels of salivary LDH level were significantly higher in head and neck cancer and OSF than in the healthy group. It is proven that salivary LDH is a noninvasive, cost-effective, and well-accepted technique that has the potential to be used as a biomarker for screening and early detection.

In OSF patients, serum and salivary LDH levels are elevated for the following reasons: First, oral epithelial tissue was lack of oxygen. Tilakarathne et al^[27]^ showed that Increased hypoxia is an essential step in the malignant transformation of OSF. In the presence of hypoxia, pyruvate is converted to lactate by a glycolytic reaction mediated by LDH. Thus, the level of LDH would increase with increasing glycolytic activity. Second, LDH activity may be associated with muscle fatigue caused by chewing betel nut. Muscle fatigue leads to tissue hypoxia, which leads to the accumulation of pyruvate, when pyruvate converted to lactate, results in high glycolytic activity. Along with the increase of glycolytic activity, LDH in some tissues will also increase.^[28]^ Muralidhar M et al^[24]^ reported that the serum LDH level was significantly higher than the normal level in pre-malignant and malignant cases.

LDH plays an important role as a marker for patients with OSF, which is reflected as follows. Firstly, when serum or salivary LDH levels change, it can be used as an effective marker for the diagnosis of patients with OSF.^[7]^ Secondly, the change in LDH levels can reflect the tissue cell damage,^[29]^ providing important information on the pathological and physiological processes of patients with OSF. Finally, during the treatment process of patients with OSF, the level of LDH may change accordingly, and the change in LDH level can be used as one of the indicators to evaluate the effectiveness of treatment.^[30]^

The study from Mishra et al^[20]^ showed that there is a direct relationship between serum LDH levels and habitual frequency of mouth opening in OSF patients Shetty et al^[28]^ found that the expression level of LDH in the saliva of males was significantly higher than that of females.

Some results were highly heterogeneous. Given the limited literature, subgroup analysis or regression analysis is not feasible, we used a random-effects model, but there was still heterogeneity. The significant heterogeneity might be attributed to some factors. Firstly, the age range of patients in the included studies was different, there were 18 to 60 years old in 1 study^[22]^ and 20 to 70 years old in the other study.^[19]^ Secondly, the gender ratio of patients included in the studies was different, with 1 study^[20]^ included only male patients, while the other study^[21]^ included both male and female patients. Finally, there was a lack of consistent and standardized procedures for collecting, storing, and measuring saliva samples. Some studies^[22]^used stimulated saliva, while others^[15,19–21]^ used unstimulated saliva. Some studies^[19–22]^ collected saliva samples all at once, while another^[15]^ collected saliva samples several times and then mixed them. One study^[20]^ measured within 12 hours after saliva collection, while another study^[15]^ measured within 24 hours after collection. All of these factors might lead to heterogeneity. Despite these limitations, this is the first meta-analysis to provide comprehensive evidence on the association between salivary and serum LHD levels in OSF patients. Therefore, this analysis is of great value and significance. In the future, we need to conduct some well-designed and larger sample size studies considering age, gender, genetic polymorphism and disease severity to obtain more reliable and valuable research results.

## 5. Conclusion

The results of our meta-analysis showed that the serum and salivary levels of LDH in OSF were higher than in healthy control. Despite the limitations of this meta-analysis, there is evidence that measurement of serum and salivary LDH can be an effective method for screening OSF. It is suggested that the determination of LDH in saliva and serum is helpful to make a better diagnosis.

## Authors contributions

**Conceptualization:** Jincai Guo.

**Data curation:** Jincai Guo.

**Formal analysis:** Tongqiang Chen.

**Funding acquisition:** Hui Xie, Jincai Guo.

**Investigation:** Hui Xie.

**Methodology:** Xueru Chen, Jincai Guo.

**Project administration:** Hui Xie.

**Resources:** Xueru Chen, Jincai Guo.

**Supervision:** Jincai Guo.

**Validation:** Hui Xie, Jincai Guo.

**Writing – original draft:** Xueru Chen.

**Writing – review & editing:** Xueru Chen, Jincai Guo.
